# Fibroids in obstructed hemivagina and ipsilateral renal anomaly‐like syndrome: Successful hysterectomy and vaginal septoplasty in a kidney transplant recipient with uterus didelphys, vaginal septum and renal agenesis

**DOI:** 10.1002/ijgo.70806

**Published:** 2026-01-13

**Authors:** Mariana Correia Moreira Cruz, Sophia Helena Batalha, Vitor Matheus Silva, Marcos de Lorenzo Messina, José Maria Soares Junior, Edmund Chada Baracat

**Affiliations:** ^1^ Obstetrics and Gynecology Department, Hospital das Clinicas HCFMUSP, Faculdade de Medicina Universidade de Sao Paulo Sao Paulo Brazil; ^2^ School of Medicine, Faculdade de Medicina FMUSP Universidade de Sao Paulo Sao Paulo Sao Paulo Brazil

**Keywords:** fibroids, hysterectomy, kidney transplant, renal agenesis, uterus didelphys, vaginal septum

Müllerian duct anomalies (MDAs) are congenital malformations affecting the fallopian tubes, uterus, and/or vagina, resulting from developmental alterations of the paramesonephric ducts.^1^ Among these anomalies, uterus didelphys is frequently associated with vaginal septum and urinary tract abnormalities, with unilateral renal agenesis being the most common.^1^, ^2^ Although compensatory mechanisms, such as kidney hypertrophy and increased filtration, help to maintain the function, individuals with a solitary kidney remain at risk for glomerular hypertension, proteinuria, and progressive renal dysfunction over time.^3^ Uterine fibroids, the most common benign tumors of the female reproductive system, typically occur in women between 30 and 50 years of age, with up to 25% of cases becoming symptomatic and significantly affecting quality of life.^4^ The most common presentation includes abnormal uterine bleeding (metrorrhagia) and dysmenorrhea, often associated with submucosal and intramural fibroids.^4^ Surgical intervention is considered when medical treatment fails, symptoms severely affect daily activities, anemia becomes refractory to medical therapy, fibroids grow rapidly, or malignancy is suspected.^4^ Hysterectomy is considered a definitive treatment option for patients who do not desire future pregnancies, offering a permanent solution for fibroid‐related symptoms.^4^

A 43‐year‐old female patient presented to our gynecology clinic with complaints of abnormal uterine bleeding and severe dysmenorrhea. Her medical history includes uterus didelphys (red circle in Figure 1a,b), a non‐obstructive longitudinal vaginal septum (Figure 1c), and unilateral renal agenesis. Thirteen years previously, the patient underwent renal transplantation (red arrow in Figure 1b) due to focal segmental glomerulosclerosis, which progressed to chronic renal failure. Over the subsequent years, the patient began experiencing progressively heavier and prolonged menstrual bleeding, lasting up to 15 days, requiring multiple pad changes daily, and leading to iron‐deficiency anemia, associated with intense cyclic pelvic pain, significantly affecting her quality of life.

**FIGURE 1:**
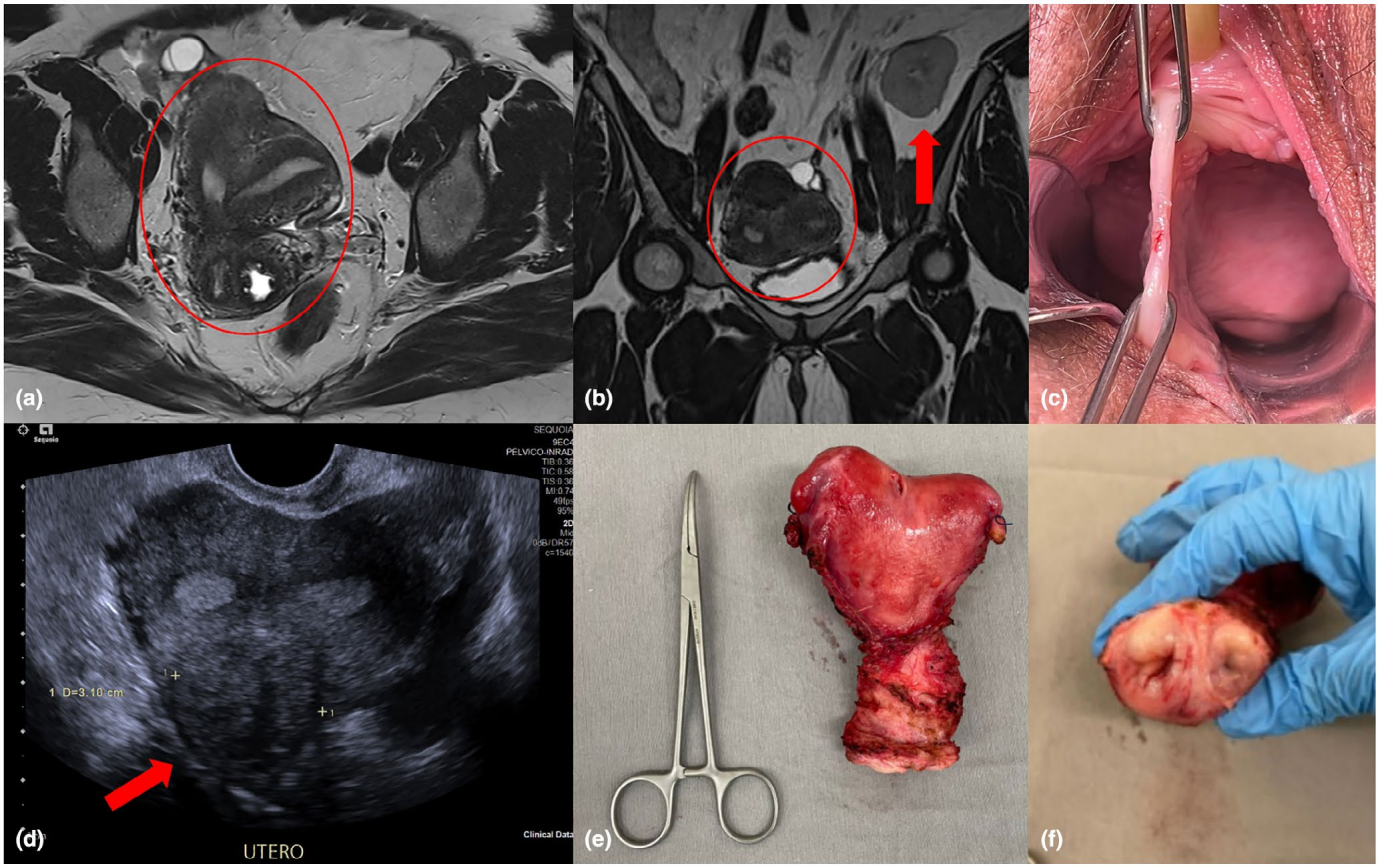
(a) Didelphic uterus (red circle) in an axial cut of MRI. (b) Didelphic uterus (red circle) and transplanted left kidney (red arrow) in a coronal cut of MRI. (c) Non‐obstructive longitudinal vaginal septum. (d) Didelphic uterus and fibroids (red arrow) in an axial cut of a transvaginal ultrasound. (e) Anatomic view of didelphic uterus. (f) Inferior view of didelphic uterus. MRI, magnetic resonance imaging.

Transvaginal ultrasound confirmed an enlarged didelphic uterus with multiple fibroids (Figure 1d) and bilateral hydrosalpinx, supporting the clinical diagnosis. The right uterine horn had a volume of 117 cm^3^, while the left measured 79 cm^3^. The largest fibroids included a 3.3 × 2.9 × 3.1 cm lesion in the posterior uterine wall (FIGO 5) (red arrow on Figure 1d), accompanied by a 1.5 × 1.0 × 1.2 cm fibroid in the lateral right uterine wall (FIGO 4), and a 1.0 × 0.9 × 0.9 cm one in the uterine fundus (FIGO 4). Initial therapeutic approach with norethisterone and tranexamic acid was attempted but poorly tolerated and ineffective. GnRH agonists were discussed as an alternative; however, the patient declined due to concerns about potential side effects. Given the failure of conservative management, the significant negative impact on the patient's quality of life and daily activities, and her decision not to pursue future pregnancies (having two living children delivered via cesarean section), total abdominal hysterectomy with bilateral salpingectomy and resection of the longitudinal vaginal septum was recommended as definitive treatment. The patient declined myomectomy and uterine artery embolization, as she no longer had reproductive desire. The abdominal hysterectomy was chosen due to the surgical team's expertise with this procedure. While laparoscopic and vaginal hysterectomies were considered, the team felt that the abdominal approach would provide the best access and control based on the specifics of the case.

The patient was cleared for surgery by the transplant team, with no additional intraoperative care instructions provided, as the renal graft function was adequate with the use of prescribed medications (tacrolimus, chlorthalidone, losartan, mycophenolate, and prednisone) and appropriate follow‐up with the renal transplant team. Surgical procedure was performed without intraoperative complications (Figure 1e,f). Postoperatively, renal function temporarily declined probably due to hypovolemia during surgery, with creatinine rising from 0.99 to 3.73. After appropriate hydration, discontinuation of antihypertensive medications, and exclusion of obstructive causes, renal function improved without the need for dialysis. Additionally, the patient evolved with a surgical wound infection 48 h after surgery, which required broad‐spectrum intravenous antibiotics (vancomycin and piperacillin/tazobactam). Despite these challenges, recovery progressed well, and the patient was discharged in a stable condition on postoperative Day 14.

This article describes a successful surgical treatment in a kidney‐transplanted patient with an obstructed hemivagina and ipsilateral renal anomaly (OHVIRA)‐like syndrome and fibroids. Surgical management in patients with uterine anomalies and kidney transplants poses unique challenges due to distorted pelvic anatomy and the need to protect the renal allograft. The graft, usually placed in the iliac fossa, often lies close to the uterus, raising the risk of inadvertent injury to the kidney or ureter. Comprehensive preoperative imaging and coordination with the transplant team are essential to delineate the graft and ureteral course.^5^ Immunosuppression further increases infection risk and delays wound healing, requiring careful perioperative planning and prophylaxis, with antibiotics and chlorhexidine‐based skin preparation.^5^ Intraoperative collaboration with transplant and anesthesia teams is also vital to manage drug interactions and maintain hemodynamic stability through fluid management.^6^ This report highlights the importance of these surgical considerations in managing similar complex scenarios, to prevent the complications previously described.

To the best of our knowledge, no similar cases have been reported in the scientific literature. This case provides insight into the successful management of fibroids and related complications in patients with OHVIRA‐like anomalies and a transplanted kidney, demonstrating that abdominal hysterectomy remains a viable treatment option.

The patient provided consent for publication, and Institutional Ethics Committee approval was not needed in HCFMUSP because the study is a case report involving a single individual, with no prospective data collection or intervention, in accordance with the institution's ethical guidelines.

## AUTHOR CONTRIBUTIONS

Mariana Correia Moreira Cruz and Sophia Helena Batalha were involved in chart review, analysis, and preparation of the manuscript. Vitor Matheus Silva was involved in study design, analysis and preparation of the manuscript. Marcos de Lorenzo Messina, José Maria Soares Junior and Edmund Chada Baracat were involved in study conceptualization, data curation and analysis, project administration, manuscript preparation, and supervision. All authors approve the final version to be published and agree to be accountable for all aspects of the work.

## FUNDING INFORMATION

This study was not funded by anyone or any organization.

## CONFLICT OF INTEREST STATEMENT

The authors have no conflicts of interest declare.

## Data Availability

Research data is not shared.
